# Anatomical Positions of Mesially/Horizontally Impacted Mandibular Third Molars are Significant Predictors for Distal Caries in Adjacent Second Molars

**DOI:** 10.1155/2022/8482209

**Published:** 2022-03-10

**Authors:** Son Hoang Le, Nhut Minh Nguyen, Ngoc Thi-Bao Nguyen, Ly Thi-Bich Nguyen

**Affiliations:** Department of Oral Surgery, Faculty of Odonto-Stomatology, University of Medicine and Pharmacy at Ho Chi Minh City, Ho Chi Minh City, Vietnam

## Abstract

**Background:**

Prevalence of distal caries in mandibular second molars (M2Ms) and its relationship with impacted condition of the adjacent mandibular third molars (M3Ms) have been reported in some studies. The results, however, were ambiguous because of including all impaction types and using univariate analysis for statistics.

**Aim:**

This study aimed to determine anatomical features of mesially/horizontally impacted mandibular third molars (M3Ms) that could predict distal caries in the adjacent mandibular second molars (M2Ms) using multivariable analysis.

**Materials and Methods:**

The study sample consisted of 300 digital panoramic radiographs of patients who underwent impacted M3Ms extraction. Two independent researchers collected the following variables from 446 pairs of M2M-M3M: sex, age, status of distal caries in M2Ms, mesial angulation, and Pell–Gregory classification of M3Ms.

**Results:**

The prevalence of distal caries was 50.67%. Multivariable Firth's logistic regression analysis showed that age (*β* = 0.066, 95% CI = 0.023–0.113), mesial angulation (<30°: *β* = −1.205, 95% CI = −1.955 to −0.499; >70°: *β* = −0.730, 95% CI = −1.184 to −0.282), vertical position (level B: *β* = 2.275; 95% CI = 0.015–7.175; level A: *β* = 3.008; 95% CI = 0.755–7.905), and horizontal position (level II: *β* = 1.515; 95% CI = 0.444–2.874; level I: *β* = 1.423; 95% CI = 0.283–2.825) were significant variables after adjusting for sex in the final model for predicting distal caries (*p* < 0.05).

**Conclusions:**

In conclusion, anatomical positions of impacted M3Ms, such as mesial angulation and Pell–Gregory classification were significant predictors of distal caries in M2Ms.

## 1. Introduction

Impacted mandibular third molars (M3Ms) are a popular oral condition, with an average prevalence of 24.4% in adults [1]. They cause numerous pathological problems in the surrounding structures, such as dental caries, periodontal pockets, pericoronitis, dental tumors, or cysts. These lesions are usually mild symptomatic or asymptomatic, and challenging to diagnose with conventional radiographs [2, 3]. Among them, distal caries in mandibular second molars (M2Ms) is one of the most common M3Ms-associated complications which range from 12.2% to 39.0% [4, 5].

Distal caries is not only popular in M2Ms but also frequently unnoticed. It has been reported that nearly one-fourth of M2Ms with distal caries are unrestorable and must be removed [6]. Consequently, it has received great attention from researchers with the aim of identifying predictors of distal caries in M2Ms adjacent to impacted M3Ms. Determining predictors would help dental clinicians anticipate the risk of distal caries and make appropriate decisions to prevent it.

There have been a number of studies regarding this topic. Most of the studies highlighted the importance of anatomical positions of the adjacent impacted M3Ms in prevalence of distal caries in M2Ms. However, the current results are limited because of the following two issues. First, majority of previous studies included all the impacted directions of M3Ms. However, the prevalence of distal caries was significantly higher in M2Ms adjacent to mesially/horizontally impacted M3Ms [7]. Second, previous studies used univariate analysis to examine the relationships between distal caries and potential factors. Because dental caries is the consequence of numerous factors, including tooth, time, bacteria, and other sociodemographic characteristics, it would be more accurate to use multivariable analysis to adjust for their contributions [8]. One study showed that the correlation between age and distal caries became insignificant after adjusting for other factors using multivariable analysis [9]. Therefore, this study was conducted using a different approach to avoid these limitations.

This study aimed to determine anatomical features of the mesially/horizontally impacted M3Ms that could predict distal caries in the adjacent M2Ms, using multivariable statistical analysis.

## 2. Materials and Methods

This cross-sectional study was conducted on 300 digital oral panoramic radiographs stored at the Department of Oral Surgery, Faculty of Odonto-Stomatology, University of Medicine and Pharmacy at Ho Chi Minh City. Radiographs were obtained from patients who underwent impacted M3Ms extraction from January 2019 to April 2021. All radiographs were taken by a sole radiographic technician using the same X-ray machine (Sirona, Germany) and technical parameters to reduce variation.

The inclusion criteria for panoramic radiographs were as follows: (1) being qualified with clear details; (2) having at least one mesially/horizontally impacted M3M; and (3) containing all of the following information: sex, birthday, and the day taken. The exclusion criteria were as follows: (1) loss of the adjacent M2M, (2) severe destruction of M2M or M3M morphology that mesial angulation and Pell-Gregory classification could not be assessed, and (3) presence of uncommon pathological signs, such as radiolucent lesions with diameter ≥2.5 mm or radiopaque lesions in the mandibular angle region.

**Figure 1:**
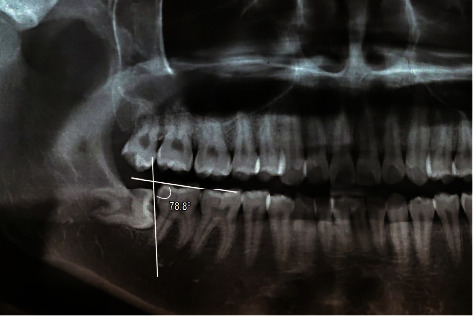
A left M2M without distal caries and method to measure mesial angulation of the impacted M3M. The mesial angulation was determined by the intersection between occlusal planes of M2M and M3M.

**Figure 2:**
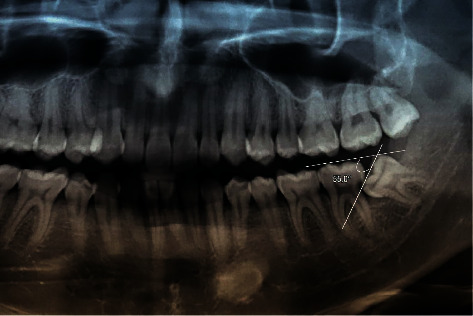
A right M2M with distal caries and mesial angulation of the adjacent M3M.

Two independent researchers (S. H. L. and N. M. N.) assessed the digital radiographs to obtain the required variables: age, sex, side (left/right), mesial angulation, Pell–Gregory classification of impacted M3Ms, and status of distal caries in M2Ms. Demographic variables (age, sex, and side) were collected from digital panoramic radiographs. The mesial angulation of impacted M3Ms was measured on a computer screen using the IC Measure software version 2.0. The researchers traced the line of the occlusal planes of M2M and M3M. The mesial angulation of the impacted M3Ms was defined as the angle formed at the intersection of the traced lines. The final mesial angulation was calculated as the mean of the values measured by the two researchers (Figures 1 and 2). The Pell–Gregory classification contained two perspectives of the impacted M3M position, including the mandibular occlusal surface (vertical position) and ramus (horizontal position). Each position has three impacted levels described and illustrated in the original article [10].

For mesial angulation, the intraclass correlation coefficient (ICC) was calculated to confirm interexaminer reliability (ICC = 0.904, 95% CI = 0.881–0.923). For the other variables (nominal variables), if there were any disagreements in the obtained values, the third researcher (L. T. B. N.) would be asked for discussion to obtain the final ones.

All data were analysed using IBM SPSS Statistics for Windows, version 25.0. Shapiro–Wilk test showed that age was not normally distributed (*p* < 0.001). A chi-squared test was employed to test differences in the prevalence of distal caries between sexes, sides, mesial angulation, and horizontal and vertical positions of impacted M3Ms. Mann–Whitney *U* test was used to examine differences in age between the caries and noncaries groups. Previous studies have shown that distal caries in M2Ms is correlated with multiple factors, such as sex, mesial angulation, and Pell–Gregory classification [9, 11, 12]. Therefore, multivariable Firth's logistic regression analysis was conducted to determine the correlation between potential factors and distal caries in M2Ms. Statistical significance was set at *p* value < 0.05.

## 3. Results

In this study, there were 446 pairs of M2M-M3M, with an average age of the samples as 24.63 years old (SD = 4.98). The prevalence of distal caries in M2Ms was 50.67%. The frequencies and prevalence of collected variables are shown in Table 1. Significant difference was found in age between the caries and noncaries group (25.35 ± 5.62 vs. 23.88 ± 4.11, respectively) with *U* = 21197.00 and *p* = 0.007. The prevalence of distal caries in M2Ms was significantly different between the groups with mesial angulation, and vertical and horizontal impaction (Table 1). Notably, dental caries in M2Ms was more frequent if the mesial angulation was 30°–70°. The prevalence of distal caries tended to increase in the less impacted M3Ms, both vertically and horizontally.

Univariate regression showed that age, mesial angulation, and vertical and horizontal impaction could be significant factors for predicting distal caries in multivariable analysis. Although sex was not a significant factor, the statistical results showed that sex was suitable for inclusion as a potential predictor in the multivariable regression test (Table 2).

Multivariable regression showed that age, mesial angulation, and vertical and horizontal impaction were significant predictors of distal caries in the final model. M2Ms adjacent to mesially/horizontally inclined M3Ms in participants of older age were more likely to suffer from distal caries. The M3Ms mesially inclined at 30°–70° increased the prevalence of distal caries in M2Ms. For vertical impaction, M3Ms level C was less related to distal caries in M2Ms than levels B and A. For horizontal impaction, M3Ms levels I and II significantly increased the probability of distal caries in M2Ms compared to level III (Table 3).

## 4. Discussion

Some studies have examined the relationship between distal caries in M2Ms and impacted M3Ms. Notably, the prevalence of distal caries in M2Ms adjacent to mesially/horizontally impacted M3Ms was usually reported as the highest among all types of impaction and could be up to nearly 10 times more than others [7, 13]. A few studies have focused on mesially/horizontally impacted M3Ms; however, multivariable analysis was not employed to adjust for contribution of independent factors [11, 14, 15]. The study results revealed that anatomical positions of mesially/horizontally impacted M3Ms, such as mesial angulation, and vertical and horizontal levels were significant predictors of distal caries in M2Ms.

The study used panoramic radiographs to assess distal caries in M2Ms and the anatomical position of M3Ms. It was also the most common radiographic technique chosen in previous studies [7]. Panoramic radiography is a reliable method for examining the anatomical characteristics of M3Ms and surrounding structures, but it is not as accurate as bitewing radiography for diagnosing proximal caries [16, 17]. However, it has been proven that the accuracy of panoramic radiography when examining proximal caries in the mandibular molar region is clinically useful and suitable [18, 19]. Therefore, panoramic radiography is appropriate for assessing distal caries in M2Ms and the anatomical position of M3Ms in this study.

Dental caries is the consequence of four direct contributors, including tooth, diet, bacteria in the biofilm, and time. Notably, bacteria in biofilms play a critical role because the acidic by-products of fermentation initiate carious lesions [8]. Evidence revealed the frequent detection of *Streptococcus*, *Lactobacillus*, *Actinomyces*, and certain species in the development of dental caries [20, 21]. Additionally, the number of dental caries-associated bacterial clusters gradually reduced when dental plaque was located in deeper positions [22, 23]. Therefore, the authors assumed that dental plaque located more apically was less able to initiate a carious lesion.

In this study, mesial angulation was divided into three groups: < 30°, 30°–70°, and >70°. The result of the effect of mesial angulation was in line with previous studies. When the mesial angulation ranged from 30° to 70°, the risk of distal caries was statistically higher than when it was <30° or >70° [9, 11, 14, 24]. To the authors' knowledge, there has been no explanation for this difference. The authors assumed that when the mesial angulation was <30°, the position of the contact point was closer to the occlusal surface; hence, it would be easier to remove dental plaque. On the other hand, when the mesial angulation was >70°, the contact point was more apical. This was not favoured for the growth of dental caries-associated bacteria [22, 23]. Perhaps, these conditions resulted in a lower prevalence of distal caries when the mesial angulation was out of the range of 30°–70°.

The Pell–Gregory classification is one of the most common predictors of distal caries in M2Ms adjacent to impacted M3Ms. The multivariable statistics revealed that in comparison with levels C and III, the other levels significantly increased the prevalence of distal caries in M2Ms. The majority of previous studies also showed the same correlations between impacted levels of M3Ms and prevalence of distal caries in M2Ms [5, 11, 12, 24–27]. According to the Pell–Gregory classification, the more impacted M3Ms were covered more by periodontal tissue; hence, the contact point of M2M and a mesially/horizontally impacted M3M was less likely to be exposed to the oral cavity [10]. This unexposed condition helped reduce the accumulation of food debris. This assumption was supported by results of previous studies which indicated a very low prevalence of distal caries in M2Ms adjacent to impacted level C and III M3Ms [5, 12, 25, 27]. Additionally, the authors assumed that the bacterial complex would shift to a smaller number of acid-producing bacterial clusters along with an increase in impacted levels [22, 23].

The results of multivariable analysis also showed that age was a significant predictor of distal caries in M2Ms. The increased remaining time of mesially/horizontally impacted M3Ms prolongs the presence of dental plaque and results in distal caries. This is in line with previous studies, with the prevalence of distal caries reportedly increased in the aging population, and the average age of participants with distal caries was significantly higher than that of their counterparts [9, 11, 14]. However, dividing participants into groups of different ages would make the previous results less relevant to clinical applications. Therefore, this study strongly supports the effect of age, with a more convincing and clinically meaningful result.

Regarding participant sex, the study found no significant correlation between sex and distal caries in M2Ms. To the authors' knowledge, only one previous study showed that the risk ratio of distal caries in males was about three times higher than that in females [9]. However, the absence of adjustment to other significant univariable factors, such as the Winter and Pell–Gregory classification, made this result questionable. In addition to the four direct contributors, dental caries is affected by personal factors such as sociodemographic status, behaviour, attitude, and knowledge [8]. Sex has been proven to affect the status of dental caries due to differences in biological and sociodemographic factors [28]. Although females have some disadvantages related to biological factors, males are less likely to have adequate oral self-care and professional treatment [29, 30]. Previous studies have reported no correlation between DMF/DMFT and the prevalence of distal caries in M2Ms adjacent to impacted M3Ms [6, 15, 31]. These results suggest that common factors for predicting high-rated dental caries, such as sex, might not have critical effects on distal caries in M2Ms. The authors assumed that distal caries in M2Ms adjacent to mesially/horizontally impacted M3Ms was mainly caused by the frequent presence of the local dental plaque that was almost impossible to clean. Bacteria in dental plaque might relate to numerous clinical signs, which appear in about 60% of cases coming for surgical removal of mesially/horizontally impacted M3Ms, such as pericoronitis, pain, or periodontal pocket [24, 31]. The higher prevalence of distal caries in mesially/horizontally impacted M3Ms than in distally and vertically ones in previous studies also support this assumption [5, 9, 12, 13, 27, 31, 32].

Previous studies examined other parameters for predicting distal caries, such as the Leone classification and the position of the M2M-M3M contact point [11, 15, 31]. The Leone classification was determined based on the distance between the cementoenamel junctions of M2M-M3M, which seemed highly correlated with the mesial angulation of the M3Ms. The higher the mesial angulations, the greater the distance between the cementoenamel junctions of M2M-M3M. However, mesial angulation has been proven to be more reliable for predicting distal caries [9, 11, 24]. In previous studies, the position of the M2M-M3M contact point was classified as above, on, and under the M2M cementoenamel junction. The authors assumed that with a same mesial angulation of M3M, the position of the M2M-M3M contact point would likely correlated with vertical impaction of the Pell–Gregory classification, and was also a parameter of the vertical impacted level of M3M [14]. In cases of advanced distal caries, the authors found that it was difficult to determine the relationship between contact points and M2M cementoenamel junctions due to the severe destruction of the tooth structure. Therefore, the Leone classification and the position of the contact point were not employed for predicting distal caries in this study.

The study was conducted with an appropriate study design: assessing digital panoramic radiographs, collecting data by two independent researchers with high interexaminer reliability, and analysing data with multivariable statistics. Notably, multivariable analysis highlighted the outstanding point because it helped reduce the confounding effects of the predictive factors. However, there are a few limitations that should be considered in future studies. First, the prevalence of distal caries would have been more accurate if it had been assessed clinically or with a three-dimensional imaging technique to avoid superimposition [33]. Second, this study did not assess the bacterial component of dental plaque, which is the most convincing answer for some of the authors' assumptions. Third, because M3M impaction, especially vertical position, can change with increasing age, a longitudinal study design would be more appropriate to predict distal caries [34]. Therefore, other researchers can consider eliminating these limitations when conducting studies to provide a better understanding.

## 5. Conclusions

This study revealed that distal caries in M2Ms could be predicted by anatomical positions of the adjacent M3Ms. Specifically, M2Ms were assessed as high-risk for distal caries if the anatomical position of the adjacent M3Ms had the following features: mesial angulation ranged from 30° to 70°, vertical impacted level A/B, and horizontal impacted level I/II. Additionally, the long presence of mesially/horizontally impacted M3M increases the likelihood of distal caries. These predictors were dependent on each other and summed up for predicting distal caries in M2Ms.

## Figures and Tables

**Table 1:** Demographic and radiographic characteristics of samples (*N*  = 446).

Variables	Caries		Noncaries		Total		*χ* ^2^	Df	*p* value
	n	%	n	%	n	%			
Sex									
Female	92	47.18	103	52.82	195	43.72	1.691	1	0.193
Male	134	53.39	117	46.61	251	56.28			
Side									
Left	102	48.57	108	51.43	210	47.09	0.701	1	0.402
Right	124	52.54	112	47.46	236	52.91			
Mesial angulation									
<30⁰	13	29.55	31	70.45	44	9.86	30.242	2	<0.001
30⁰–70⁰	159	61.63	99	38.37	258	57.85			
>70⁰	53	36.81	90	63.19	144	32.29			
Vertical impaction									
A	167	60.95	107	39.05	274	61.44	36.255^*∗*^	2	<0.001
B	59	36.42	103	65.58	162	36.32			
C	0	0	10	100	10	2.24			
Horizontal impaction									
I	64	56.63	49	43.37	113	25.34	20.800	2	<0.001
II	159	52.30	145	57.70	304	68.16			
III	3	10.34	26	89.66	29	6.50			

A chi-squared test was conducted to compare differences between caries and noncaries groups, except for the vertical impaction groups. Owing to the appearance of expected values less than 5, Fisher's exact test was conducted.^*∗*^F-value of Fisher's exact test.

**Table 2:** Univariate logistic regressions of associations between distal caries in M2Ms and demographic/radiographic characteristics (*N*  = 446).

Variables	Coefficient	SE	95% CI	*p* value
Sex^a^	0.247	0.191	−0.126–0.623	0.194
Age	0.063	0.021	0.023–0.106	0.002
Mesial angulation^b^				
<30°	−1.319	0.353	−2.033–−0.653	<0.001
>70°	−0.979	0.214	−1.402–−0.563	<0.001
Vertical impaction^c^				
B	2.491	1.527	0.410–7.354	0.013
A	3.488	1.523	1.421–8.349	<0.001
Horizontal impaction^d^				
II	2.116	0.590	1.102–3.445	<0.001
I	2.289	0.609	1.229–3.646	<0.001

SE, standard error; CI, confidence interval; ^a^0 = female; 1 = male; ^b^1 = 30°–70°; 2 = <30°, 3 = >70°; ^c^1 = C; 2 = B; 3 = A; ^d^1 = III; 2 = II; 3 = I.

**Table 3:** Multivariable logistic regression of associations between distal caries in M2Ms and demographic/radiographic characteristics (*N*  = 446).

Variables	Coefficient	SE	95% CI	*p* value
Sex^a^	0.057	0.212	−0.358–0.470	0.788
Age	0.066	0.023	0.023–0.113	0.002
Mesial angulation^b^				
<30°	−1.205	0.373	−1.955–−0.499	0.001
>70°	−0.730	0.231	−1.184–−0.282	0.001
Vertical impaction^c^				
B	2.275	1.613	0.015–7.175	0.048
A	3.008	1.611	0.755–7.905	0.005
Horizontal impaction^d^				
II	1.515	0.606	0.444–2.874	0.004
I	1.423	0.637	0.283–2.825	0.013

SE, standard error; CI, confidence interval. ^a^0 = female, 1 = male; ^b^1 = 30⁰–70⁰, 2 = <30⁰, 3 = >70⁰; ^c^1 = C, 2 = B, 3 = A; ^d^1 = III, 2 = II, 3 = I.

## Data Availability

All the data used to support the findings of this study were supplied by Dr. Son Hoang Le under license and so cannot be made freely available. Requests for access to these data should be made to Dr. Son Hoang Le.
